# Allogeneic CAR T cells for autoimmune diseases: a glimpse into the future

**DOI:** 10.1038/s41392-024-01998-8

**Published:** 2024-10-08

**Authors:** Dimitrios Mougiakakos

**Affiliations:** Department of Hematology, Oncology, and Cell Therapy, Otto-von-Guericke University Magdeburg, Magdeburg, Germany

**Keywords:** Medical research, Rheumatology, Drug development

In a recent study published in *Cell*, Wang X et al.^1^ reported the first use of allogeneic anti-CD19 CAR T cells in patients with therapy-resistant autoimmune diseases, demonstrating their effectiveness in reducing disease activity, with good tolerability and persistence. These findings suggest that allogeneic CAR T cells could offer a scalable, off-the-shelf treatment option for autoimmune disorders.

The first successful treatment of a patient with refractory systemic lupus erythematosus using autologous anti-CD19 CAR T cells^2^ has sparked interest in the repurposing of CAR T cells in autoimmune diseases,^3^ and this study raises the question of the benefits of an allogeneic approach. A key benefit is the potential for improved accessibility to therapy. In the U.S. and Europe, 5–10% of the population suffers from an autoimmune disease, meaning that a potentially large number of patients could benefit from and would seek CAR T-cell therapy. The autologous CAR T-cell products currently approved for malignant diseases are expensive, require long logistics chains, and therefore take a long time from decision to use to actual infusion. However, off-the-shelf allogeneic CAR T cells could reduce costs by eliminating the need for apheresis, reducing logistics costs, and increasing production scalability. In the study presented, cells from a single donor were sufficient to produce CAR T cells for approximately one hundred patients.

Additional strengths of allogeneic products include the use of T cells that have not been compromised by prior immunosuppressive or cytotoxic treatments and the absence of the risk of transducing autoreactive T cell clones. Off-the-shelf availability also reduces the risk of disease exacerbation due to a sudden discontinuation of immunosuppressants in order to be able to perform an autologous lymphocyte apheresis. The use of allogeneic CAR T cells is not without its specific risks; however, the authors of the study have carefully addressed several critical aspects in the design of these allogeneic products. To prevent graft-versus-host disease (GvHD), the T cell receptor (TCR) is removed by genetic ablation of the T cell receptor alpha chain constant (TRAC) gene, thereby preventing expression of the TCR/CD3 complex. To reduce allogenicity and increase the persistence of CAR T cells, expression of certain MHC class I and all MHC class II molecules is removed by knocking out HLA-A, HLA-B, and class II major histocompatibility complex transactivator (CIITA), while HLA-C, HLA-E, and HLA-G are retained to avoid NK cell-mediated elimination. PD-1 is also knocked out under the assumption that PD-L1 is often upregulated in inflamed tissues, which could lead to premature senescence of CAR T cells.

**Fig. 1:**
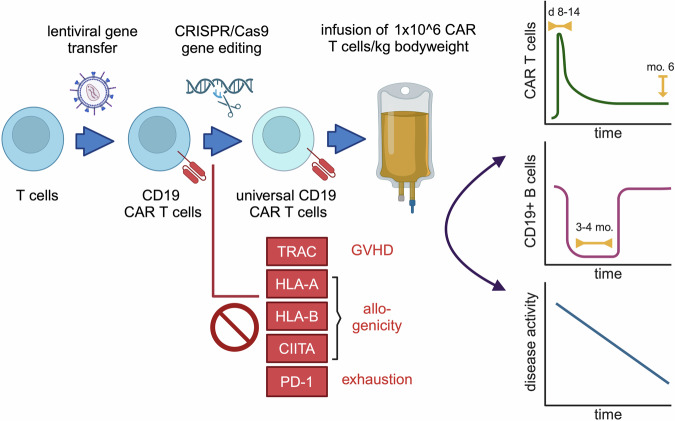
Generation and efficacy of allogeneic “universal” CD19 CAR T cells for the treatment of refractory autoimmune diseases. Healthy donor-derived T cells were transduced with a CD19 CAR-encoding lentiviral vector. CRISPR/Cas9-based gene editing was then used to ablate expression of T cell receptor alpha chain constant (TRAC), HLA-A, HLA-B, major histocompatibility complex class II transactivator (CIITA), and PD-1 to reduce the risk of graft versus host disease (GvHD), increase persistence by reducing allogenicity, and prevent premature exhaustion. Following lymphodepletion, allogeneic CAR T cells were infused into the patient, resulting in the expansion of CAR T cells that peaked between day (d) 8-14 and were still detectable at month (mo.) six, rapid but transient (for 3-4 mo.) depletion of B cells, and improvement in disease activity

Methodologically, the process begins with apheresis, followed by T cell isolation (Fig. 1). The CD19 CAR is then introduced into the T cells using a lentiviral vector. The CD19 antigen has proven to be an excellent target in autoimmune diseases thus far, due to its broad expression across B cell populations -from pro-B cells in the bone marrow to plasmablasts.^4^ The only exception are long-lived plasma cells, which means that the memory of humoral immunity is less impacted, allowing for example antiviral antibody titers to be preserved despite deep B cell depletion. Subsequently, CRISPR-Cas9-based gene editing is performed to knock out HLA-A, B, CIITA, TRAC, and PD-1. The cells are expanded for 12 days, remaining CD3+ T cells are depleted to avoid GvHD, and the final product, TyU19, is reinfused at a dose of 1 × 10^6^ CAR T cells/kg body weight after classical three-day lymphodepletion with fludarabine and cyclophosphamide. All immunosuppressants, except for steroids, are discontinued prior to infusion. Various molecular genetic analyses including whole genome sequencing, GUIDE-seq, and SITE-seq were performed to exclude high-risk insertions and increased genomic instability. The region with the strongest off-target editing was HLA-C, which can be explained by sequence homologies with HLA-A and HLA-B. This should not result in any disadvantages regarding immunogenicity and/or NK cell targeting.

To date, three patients with severe, progressive, and polyrefractory autoimmune diseases have been infused within a basket study (NCT05859997). One patient with immune-mediated necrotizing myopathy (IMNM) positive for anti-signal recognition particle (SRP) antibodies and two patients with anti-Scl-70 antibody-positive, diffuse cutaneous systemic sclerosis (SSC) were treated. IMNM is characterized by severe muscle weakness, elevated levels of muscle enzymes, and the presence of muscle fiber necrosis without significant inflammation. SSC is a progressive, hard-to-treat disease leading to widespread fibrosis of the skin and internal organs, including the lungs, heart, and kidneys, which can result in significant morbidity and mortality due to organ dysfunction. Tolerability was excellent, with no cytokine release syndrome, neurotoxicity, GvHD, prolonged cytopenia, hypogammaglobulinemia requiring immunoglobulin substitution, or severe infections. CAR T cells expanded robustly, reaching an initial peak between days 8 and 14 (Fig. 1). B-cell depletion in the peripheral blood was immediate and later confirmed in tissue biopsies from SSC patients, consistent with recent data showing that CAR T cells, unlike monoclonal antibodies, lead to B-cell depletion in inflamed tissues.^3^ Consistent with other CAR T cell studies in autoimmune diseases, the duration of B cell aplasia was transient, lasting 2-3 months.

All patients showed a significant reduction in disease activity, with partial normalization of laboratory parameters and improved quality of life, sustained at the 6-month follow-up even after B cell recovery. Pathogenic antibodies showed seroconversion in IMNM, while in SSC, they were significantly reduced or stabilized over time. Regardless, the clinical efficacy was evident, further highlighting the complex role of B cells in autoimmune diseases, which extends beyond autoantibody production to include roles as sources of pro-inflammatory cytokines and antigen-presenting cells for self-peptides. Interestingly, the persistence of the CAR T cells in all three patients was unexpectedly long, with detection at the 6-month mark, and was longer than another product that is also designed to have a limited lifespan for safety reasons, namely mRNA-based BCMA CAR T cells,^5^ which have already shown promising results in myasthenia gravis.

Overall, the data are very promising and quite comparable in terms of efficacy and tolerability to the data from the largest case series with the longest follow-up to date evaluating autologous CD19 CAR T cells in refractory autoimmune diseases.^4^ Of course, the main limitation is the small number of patients and the relatively short follow-up. The study is still recruiting, and we eagerly await more patients and longer follow-up results. The allogeneic CAR T cell concept presented here could potentially address several of the anticipated challenges in the future of cell therapy for autoimmune diseases.
